# Characterization of *N*-acylhomoserine lactone-degrading bacteria associated with the *Zingiber officinale *(ginger) rhizosphere: Co-existence of quorum quenching and quorum sensing in *Acinetobacter *and *Burkholderia*

**DOI:** 10.1186/1471-2180-11-51

**Published:** 2011-03-08

**Authors:** Kok-Gan Chan, Steve Atkinson, Kalai Mathee, Choon-Kook Sam, Siri Ram Chhabra, Miguel Cámara, Chong-Lek Koh, Paul Williams

**Affiliations:** 1 Division of Genetics and Molecular Biology, Institute of Biological Sciences, Faculty of Science, University of Malaya, 50603 Kuala Lumpur, Malaysia; 2 School of Molecular Medical Sciences, Centre for Biomolecular Sciences, University of Nottingham, Nottingham, NG7 2RD, UK; 3 Department of Molecular Microbiology and Infectious Diseases, Herbert Wertheim College of Medicine, Florida International University, Miami, FL 33199, USA; 4 Natural Sciences and Science Education AG, National Institute of Education, Nanyang Technological University, 1 Nanyang Walk, Singapore 637616, Singapore; 5 Institute of Biological Sciences (Division of Genetics and Molecular Biology), Faculty of Science, University of Malaya, 50603 Malaysia

## Abstract

**Background:**

Cell-to-cell communication (quorum sensing (QS)) co-ordinates bacterial behaviour at a population level. Consequently the behaviour of a natural multi-species community is likely to depend at least in part on co-existing QS and quorum quenching (QQ) activities. Here we sought to discover novel *N*-acylhomoserine lactone (AHL)-dependent QS and QQ strains by investigating a bacterial community associated with the rhizosphere of ginger (*Zingiber officinale) *growing in the Malaysian rainforest.

**Results:**

By using a basal growth medium containing *N*-(3-oxohexanoyl)homoserine lactone (3-oxo-C6-HSL) as the sole source of carbon and nitrogen, the ginger rhizosphere associated bacteria were enriched for strains with AHL-degrading capabilities. Three isolates belonging to the genera *Acinetobacter *(GG2), *Burkholderia *(GG4) and *Klebsiella *(Se14) were identified and selected for further study. Strains GG2 and Se14 exhibited the broadest spectrum of AHL-degrading activities via lactonolysis while GG4 reduced 3-oxo-AHLs to the corresponding 3-hydroxy compounds. In GG2 and GG4, QQ was found to co-exist with AHL-dependent QS and GG2 was shown to inactivate both self-generated and exogenously supplied AHLs. GG2, GG4 and Se14 were each able to attenuate virulence factor production in both human and plant pathogens.

**Conclusions:**

Collectively our data show that ginger rhizosphere bacteria which make and degrade a wide range of AHLs are likely to play a collective role in determining the QS-dependent phenotype of a polymicrobial community.

## Background

Bacteria employ sophisticated cell-to-cell communication networks which instigate population-wide behavioural changes in response to environment stimuli. Such population-dependent adaptive behaviour results in altered gene expression in response to the production and sensing of chemical information in the form of diffusible signal molecules, commonly referred to as autoinducers. The process, whereby an increase in the concentration of signal molecule(s) in the extracellular milieu reflects cell population density is called 'quorum sensing' (QS). At a threshold concentration of the QS signal molecule (when the population is considered to be 'quorate'), the target genes are induced or repressed. In different bacterial genera, these may include genes which code for the production of secondary metabolites, plasmid transfer, motility, virulence, and biofilm development (for reviews see [1,2]).

In many Gram-negative bacteria, QS depends on the actions of *N*-acylhomoserine lactone (AHL) signal molecules [1,2]. These consist of a homoserine lactone ring linked via a saturated or unsaturated acyl chain (generally between 4 and 18 carbons) and without or with a keto or hydroxy substituent at the C3-position (for reviews see [1,2]). AHL biosynthesis primarily depends on the actions of enzymes belonging to the LuxI or LuxM protein families while the response to an AHL is usually driven by the interaction between the signal molecule and a member of the LuxR protein family of response regulators [1,2].

Since QS controls a range of biological functions associated with virulence and as the emergence of multi-antibiotic resistant bacterial strains is in the ascendency, there is increasing pressure to discover novel therapeutic approaches to combat bacterial infections [3,4]. Interrupting QS may represent one such method which has the added advantage that the targets are not normally essential for bacterial survival and therefore are not subject to the same selective pressures observed for conventional growth inhibitory antimicrobials [3-6].

Quorum quenching (QQ) refers to the process in which QS is disrupted. QQ can be achieved in several ways such as through the enzymatic destruction of QS signal molecules, the development of antibodies to QS signal molecules or via agents which block QS. In this context both the QS signal synthase and sensor or response regulator proteins are the primary targets [3-6].

Under alkaline conditions AHLs are rapidly inactivated by pH-dependent lactonolysis in which the homoserine lactone ring is hydrolysed to the ring open form (i.e. the corresponding *N*-acylhomoserine compound) in a reaction which can be reversed by acidification [7,8]. This reaction can also be driven enzymatically by lactonases such as AiiA, AttM, AiiB [9,10] and AhlD [11]. There is also a second class of AHL-degrading enzymes which are amidases/acylases such as AiiD [12] and PvdQ [13] which cleave the AHL amide bond releasing homoserine lactone and the corresponding fatty acid.

The ability to inactivate AHLs enzymatically is shared by diverse bacteria belonging to the α-*Proteobacteria *including *Agrobacterium*, *Sphingomonas*, *Sphingopyxis *and *Bosea*, the β-*Proteobacteria *such as *Variovorax*, *Ralstonia *and *Comamonas*, the γ-*Proteobacteria *including *Pseudomonas *and *Acinetobacter*, Firmicutes such as *Bacillus *and Actinobacteria such as *Rhodococcus *as well as the *Streptomyces *sp. (reviewed by Uroz *et al *[6]).

Since QS often controls virulence in both plant and animal pathogens [1,2], QQ bacteria have potential as biocontrol agents which protect plants from pathogens while novel AHL-inactivating enzymes may have utility as therapeutic agents [6]. Consequently, we have been exploring novel rhizosphere environments for bacterial communities displaying both AHL-dependent QS and AHL-degrading activities. Since both beneficial rhizosphere bacteria and pathogens may use the same or very similar AHLs, it is important that QQ directed toward the latter do not perturb the former [6]. Hence the identification of strains expressing highly specific QQ enzymes would have significant utility. Here we focus on the AHL-inactivating activities of a community of bacteria associated with the roots of *Zingiber officinale *(ginger) growing in the Malaysian rainforest. Three AHL-inactivating bacteria belonging to the genera *Acinetobacter*, *Burkholderia *and *Klebsiella *were identified and isolated using an enrichment assay employing *N*-(3-oxohexanoyl)homoserine lactone (3-oxo-C6-HSL) as the sole nitrogen and carbon source. While the *Acinetobacter *and *Klebsiella *strains both exhibited broad spectrum lactonase activity, the *Burkholderia *strain reduced 3-oxo-AHLs to the corresponding 3-hydroxy compounds. The *Acinetobacter *and *Burkholderia *strains both possessed AHL-producing and AHL-inactivating activities and in co-culture experiments we show that all three ginger rhizosphere isolates are capable of reducing the production of key *P. aeruginosa *QS-dependent virulence determinants and *Erwinia carotovora*-mediated tissue damage in a potato tuber infection model.

## Results

### Selection of QQ bacteria from ginger rhizosphere

To enrich for rhizosphere-associated bacteria with AHL-degrading capabilities, a ginger rhizosphere suspension was used to inoculate a basal medium containing 3-oxo-C6-HSL as the sole source of carbon and nitrogen [14]. Bacterial growth was evident within 48 h but only in the samples containing 3-oxo-C6-HSL (data not shown). The enrichment culture was plated onto solidified basal KG medium [14] containing 3-oxo-C6-HSL which was passaged for single colonies which were subcultured on LB agar. Seven ginger rhizosphere-associated bacteria with four distinctive morphotypes (GG1, GG2, GG3, GG4, GG5, GGp and Se14) were chosen for further study.

The ginger rhizosphere strains were identified by 16S rDNA sequencing and analysis of the aligned sequences (1498 nucleotides) was performed by web-based similarity searches against the GenBank database. The strains were identified as *Acinetobacter *spp. (GG2 and GG3), *Burkholderia *spp. (GG1 and GG4), *Klebsiella *sp. (GG5 and Se14) and *Microbacterium *sp. (GGp). Since the 16S rDNA sequence data indicated that GG1, GG3 and GG5 are very closely related to GG4, GG2 and Se14 respectively, we chose to focus on GG2, GG4 and Se14. GGp was also omitted from further investigation. The GG2 16S rDNA sequence showed 99% identity with *Acinetobacter *spp. and clustered phylogenetically with *Acinetobacter calcoaceticus *[GenBank Accession Number EF432578] and a poorly characterized *Acinetobacter *sp. [GenBank Accession Number DQ366106]). The GG4 16S rDNA shared 99% sequence identity with *Burkholderia cepacia *PRE5 [GenBank Accession Number AY946011) while Se14 is most closely related to *Klebsiella *species PN2 [GenBank Accession Number AY946011]. The accession numbers for the 16S rDNA sequences of *Acinetobacter *sp. (GG2) [GenBank: GQ245971], *Burkholderia *sp. (GG4) [GenBank: HQ728437] and *Klebsiella *sp. (Se14) [GenBank: HQ728438] have been deposited with GenBank.

**Figure 1:**
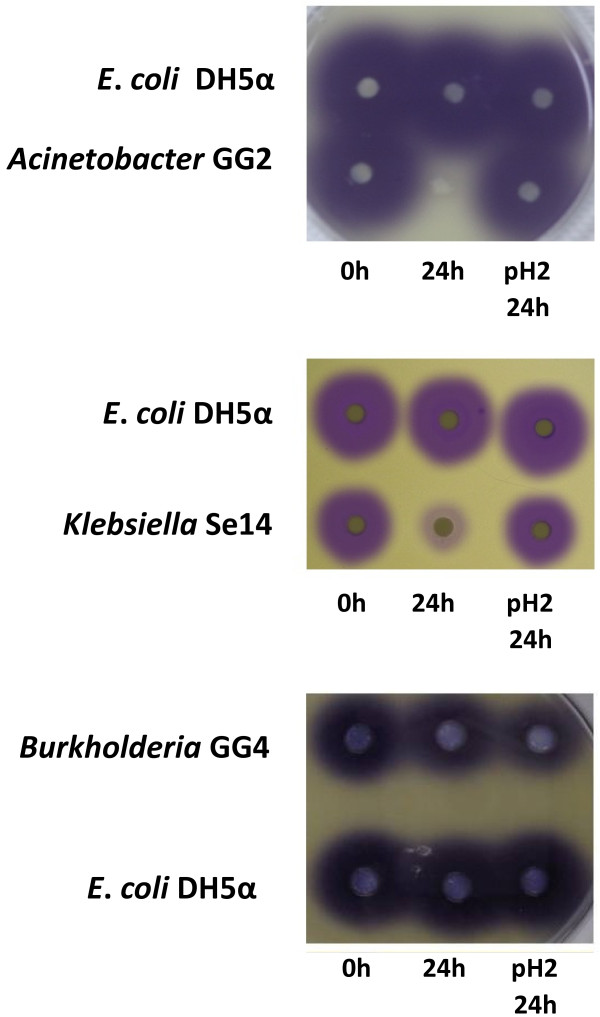
**3-oxo-C6-HSL degradation by *Acinetobacter *GG2, *Burkholderia *GG4 and *Klebsiella *Se14 quorum quenching bacteria isolated from the ginger rhizosphere**. Each rhizosphere bacterium or *E. coli *DH5α was incubated with 3-oxo-C6-HSL for 0, 24 h after which the cell culture supernatants were either spotted directly onto paper disks or acidified to pH 2 for 24 h to recyclize any ring opened 3-oxo-C6-HSL before spotting onto paper disks. These were placed on an agar plate overlaid with *C. violaceum *CV026 and incubated. A purple halo indicates the presence of 3-oxo-C6-HSL.

The 3-oxo-C6-HSL-inactivating activity of each strain was assessed, and Figure 1 shows the lack of any residual 3-oxo-C6-HSL after incubation with GG2 or with Se14 for 24 h. However, 3-oxo-C6-HSL was still detected after incubation with GG4 cells for 24 h (Figure 1).

### Characterization of QQ Activities of *Acinetobacter *GG2, *Burkholderia *GG4 and *Klebsiella *Se14

To determine the range of AHLs inactivated by each of the three ginger rhizosphere isolates, whole cells resuspended in PBS buffer were incubated for up to 96 h with a range of AHLs differing in acyl chain length (C4-C14), the presence or absence of a C3 substituent (oxo or hydroxy) or with a series of 3-hydroxy-C14-HSLs with a double bond at either C9, C10, C11 or C13 (Table 1). After incubation, any remaining AHLs were detected using the appropriate AHL biosensor as described in the **Methods **section and compared with *Escherichia coli *DH5α and PBS as negative controls. The data obtained are summarized in Table 1. Using biosensor assays *Klebsiella *Se14 inactivated all of the AHLs tested while *Acinetobacter *GG2 showed broad activity but was most effective against the long chain unsubstituted or 3-hydroxy substituted saturated or unsaturated acyl chain-AHLs (Table 1). *Burkholderia *GG4 exhibited no apparent activity against the AHLs using these biosensor assays (data not shown).

**Table 1:** AHLs degraded by GG2 and Se14

Types of AHL tested	AHL-degradation pattern	
	GG2	Se14
C4-HSL	**+**	**+ + +**
C5-HSL	**+ +**	**+ + +**
C6-HSL	**+ +**	**+ + +**
C7-HSL	**+ +**	**+ + +**
C8-HSL	**+ +**	**+ + +**
C9-HSL	**+ + +**	**+ + +**
C10-HSL	**+ + +**	**+ + +**
C11-HSL	**+ + +**	**+ + +**
C12-HSL	**+ + +**	**+ + +**
C14-HSL	**+ + +**	**+ + +**
3-hydroxy-C4-HSL	**+ +**	**+ + +**
3-hydroxy-C6-HSL	**+ +**	**+ + +**
3-hydroxy-C8-HSL	**+ +**	**+ + +**
3-hydroxy-C10-HSL	**+ + +**	**+ + +**
3-hydroxy-C12-HSL	**+ + +**	**+ + +**
3-hydroxy-C14-HSL	**+ + +**	**+ + +**
3-oxo-C8-HSL	**+ +**	**+ + +**
3-oxo-C10-HSL	**+ +**	**+ + +**
3-oxo-C12-HSL	**+ +**	**+ + +**
3-oxo-C14-HSL	**+ +**	**+ + +**
Δ^9^-3-hydroxy-C14-HSL	**+ + +**	**+ + +**
Δ^10^-3-hydroxy-C14-HSL	**+ + +**	**+ + +**
Δ^11^-3-hydroxy-C14-HSL	**+ + +**	**+ + +**
Δ^13^-3-hydroxy-C14-HSL	**+ + +**	**+ + +**

Degradation of AHLs by GG2 and Se14. Degradation of AHL: + : weak; ++ : moderate; + + + : significant. Insets denote the digital image of AHLs detected using the biosensors *E*. *coli *[pSB401] and/or *E*. *coli *[pSB1075]; evaluated according to the reduction in bioluminescence. All experiments took into account the detection limit of the biosensors used for each AHL-degradation assay.

**Figure 2:**
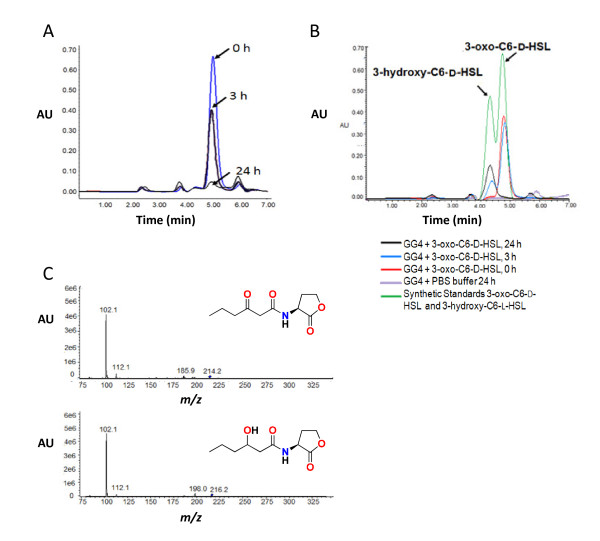
**HPLC analysis of the degradation of 3-oxo-C6-D-HSL after incubation with *Acinetobacter *GG2 and *Burkholderia *GG4**. (A) The D-isomer of 3-oxo-C6-HSL was incubated for 0- (blue line), 3- (black line) and 24 h (grey line) with GG2, the culture supernatant extracted with ethyl acetate and subjected to HPLC analysis. The data show the disappearance of the AHL peak at 5.0 min after 24 h incubation. (B) When incubated with GG4 over a period from 0- (red line), 3- (blue line) and 24 h (black line), the 3-oxo-C6-D-HSL peak is replaced by a new peak at about 4.3 min which co-migrates with 3-hydroxy-C6-HSL. The controls used were synthetic 3-oxo-C6-D-HSL, 3-hydroxy-C6-D-HSL (green line) and PBS buffer incubated with GG4 for 24 h to ensure no 3-hydroxy-C6-HSL production by GG4 (purple line). (C) MS showing the presence of 3-oxo-C6-HSL at 0 h (upper panel; *m/z *214.2 [M+H]) and 3-hydroxy-C6-HSL after 24 h (lower panel; *m/z *216.2 [M+H]) when 3-oxo-C6-L-HSL was incubated with GG4.

Since natural AHLs are in the L-configuration, we sought to determine whether the AHL inactivating activities observed were stereospecific. After incubation of GG2 and Se14 whole cells with the D-isomer of 3-oxo-C6-HSL (3-oxo-C6-D-HSL), the reaction mixture was extracted and analysed by HPLC rather than using the AHL biosensors which do not respond to D-isomers. For GG2 and Se14 the peak corresponding to 3-oxo-C6-D-HSL was reduced after 3 h incubation and effectively absent after 24 h. The data for *Acinetobacter *strain GG2 are shown in Figure 2A. Similar results were obtained for Se14 (data not shown) indicating that AHL inactivation by these two ginger rhizosphere bacteria is not stereospecific. Although the biosensor assays did not demonstrate any AHL inactivation for *Burkholderia *GG4, HPLC analysis revealed that the peak corresponding to 3-oxo-C6-D-HSL was reduced with the concomitant appearance of a new peak (Figure 2B) suggesting that GG4 was capable of modifying AHLs.

### Identification of the AHL degradation products

To determine whether *Acinetobacter *strain GG2 inactivated AHLs through cleavage of the acyl chain or via lactonolysis or both, 3-oxo-C6-HSL was first incubated with GG2 cells for 24 h. The cells were removed and the supernatant was collected, acidified to pH 2 and incubated for a further 24 h. This results in the pH-mediated re-cyclization of any ring opened compound present [8] which was subsequently detected using the *C. violaceum *CV026 AHL biosensor [15]. Figure 1 shows that while no 3-oxo-C6-HSL was detected in the supernatant after 24 h incubation with GG2, it could be recovered by acidification indicating that GG2 possesses lactonase activity. To investigate whether GG2 also exhibits amidase activity a cell-free GG2 24 h culture supernatant grown in the presence of 3-oxo-C6-HSL was treated with dansyl chloride which reacts with the exposed free amine of the homoserine lactone ring following release of the AHL acyl chain [16]. No dansylated homoserine lactone was detected indicating that GG2 does not exhibit acylase activity (data not shown).

Similar acidification experiments to those described above for *Acinetobacter *GG2 were carried out for *Klebsiella *Se14. These showed that Se14 also possesses a lactonase. Since *Klebsiella pneumoniae *has previously been reported [11] to possess a homologue of the *Arthrobacter *lactonase gene *ahlD *termed *ahlK*, we used primers based on *ahlK *to determine whether the gene was also present in Se14. A single PCR product was obtained and sequenced and found to be identical to the *ahlK *gene (data not shown). When Se14 *ahlK *was expressed in *E. coli *DH5α, AHL biosensor assays revealed that there was a reduction in bioluminescence consistent with a decrease in 3-oxo-C6-HSL when compared to the vector control. Upon acidification of the supernatant AHL biosensor activity could be restored thus confirming that AhlK has lactonase activity (data not shown).

When *Burkholderia *strain GG4 was incubated with 3-oxo-C6-D-HSL for 3 h and examined by HPLC, we noted the appearance of a new peak (retention time 4.3 min) that was absent when either GG2 or Se14 was incubated with the same D-isomer (retention time 4.8 min) (Figure 2B). Similar results were obtained following incubation of the natural L-isomer of 3-oxo-C6-HSL with GG4 and the new peak was found to co-migrate with the L-isomer of 3-hydroxy-C6-HSL (data not shown) suggesting that GG4 has oxido-reductase activity towards 3-oxo-AHLs. To confirm the oxido-reductase activity of GG4, 3-oxo-C6-L-HSL incubated with GG4 for 0 h and 24 h was analysed by mass spectrometry and the appearance of 3-hydroxy-C6-HSL was confirmed by detection of the precursor ion (*m/z *216.2 ([M+H])) and fragment ions of *m/z *198.0 ([M+H-H_2_O]) and 102.0 (Figure 2C) which are characteristic of 3-hydroxy-AHLs which readily lose a water molecule and the homoserine lactone moiety respectively [17,18].

Similar results were obtained on incubation of GG4 with the L-isomers of 3-oxo-C4-HSL or 3-oxo-C8-HSL in that new HPLC peaks co-eluting with 3-hydroxy-C4-HSL and 3-hydroxy-C8-HSL synthetic standards, respectively, were observed after incubation for 6 h (data not shown). This oxido-reductase activity was only apparent when 3-oxo-AHLs were incubated with GG4 whole cells but not cell lysates (data not shown).

### *Acinetobacter *GG2 and *Burkholderia *GG4 produce AHLs

Since some but not all *Acinetobacter *and *Burkholderia *strains have previously been reported to produce AHLs, we wondered whether QQ and QS activities co-exist in GG2, GG4 and Se14. To determine whether any of the three ginger rhizosphere strains produced AHLs, they were first cross-streaked against each of three different AHL biosensors namely *C. violaceum *CV026, *E. coli *[pSB401] and *E. coli *[pSB1075], and the plates examined over time for the induction of violacein or bioluminescence (data not shown). GG2 induced bioluminescence in *E. coli *[pSB1075] indicating that it was producing long chain AHLs, GG4 activated both CV026 and *E. coli *[pSB401] indicative of short chain AHL production while Se14 failed to activate any of the three AHL biosensors.

**Figure 3:**
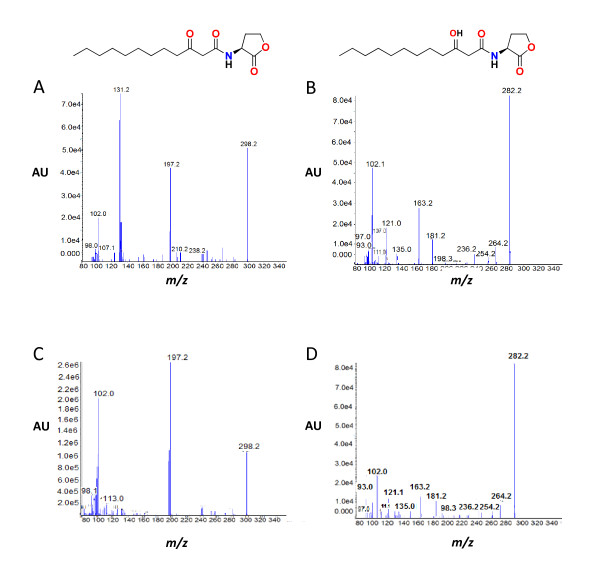
**Mass spectra of the AHLs produced by GG2**. Extracts from spent culture supernatants of GG2 were analysed by LC-MS/MS. The fragment ion at *m/z *102 is characteristic of the homoserine lactone ring (A and B). By comparison with the corresponding synthetic AHL standards (C and D) the precursor ion of *m/z *298.2 and fragment ion of *m/z *197.2 demonstrate the presence of 3-oxo-C12-HSL (A) whereas the precursor ion of *m/z *282.2 (which corresponds to [M-H_2_O]) and fragment ion of *m/z *181.2 are characteristic for 3-hydroxy-C12-HSL (B). AU: Absorbance unit.

To identify unequivocally the AHLs produced by GG2, spent culture supernatant extracts were analysed by liquid chromatography (LC) coupled to hybrid quadruple-linear ion trap mass spectrometry (LC-MS/MS), which revealed the presence of 3-oxo-C12-HSL and 3-hydroxy-C12-HSL by comparison of their retention times, precursor and principal fragment ions with synthetic standards. Figure 3 shows the fragmentation patterns for 3-oxo-C12-HSL (precursor ion *m*/*z *298.2 [M+H]; fragment ions *m/z *197.2, 102.0 (Figure 3A and Figure 3C) and 3-hydroxy-C12-HSL (precursor ion *m*/*z *282.2 [M-H_2_O]; fragment ions *m*/*z *181.2, 102.1) (Figure 3B and Figure 3D). The mass spectra of the extracted AHLs were similar to the corresponding synthetic compounds. Quantitative analysis by LC-MS/MS of the AHLs produced by GG2 over a 24 h period revealed that 3-hydroxy-C12-HSL was the most abundant AHL produced by GG2 which attains a maximum level after 12 h growth, but is almost undetectable after 24 h (data not shown).

LC-MS/MS analysis of GG4 supernatants confirmed the presence of 3-oxo-C6-HSL (precursor ion *m*/*z *214.2 [M+H]; fragment ions *m*/*z *113.0, 102.0); C8-HSL (precursor ion *m*/*z *228.2 [M+H]; fragment ions *m*/*z *109.1, 102.0), 3-hydroxy-C8-HSL (precursor ion *m*/*z *226.2 [M-H_2_O]; fragment ions *m*/*z *125.1, 102.0) and C9-HSL (precursor ion *m*/*z *242.2 [M-H_2_O]; fragment ions *m*/*z *142.2, 102.1) (Additional File 1). The mass spectra of the extracted AHLs were indistinguishable from the corresponding synthetic compounds (Additional File 1).

### QQ biocontrol activity of the ginger rhizosphere isolates

**Figure 4:**
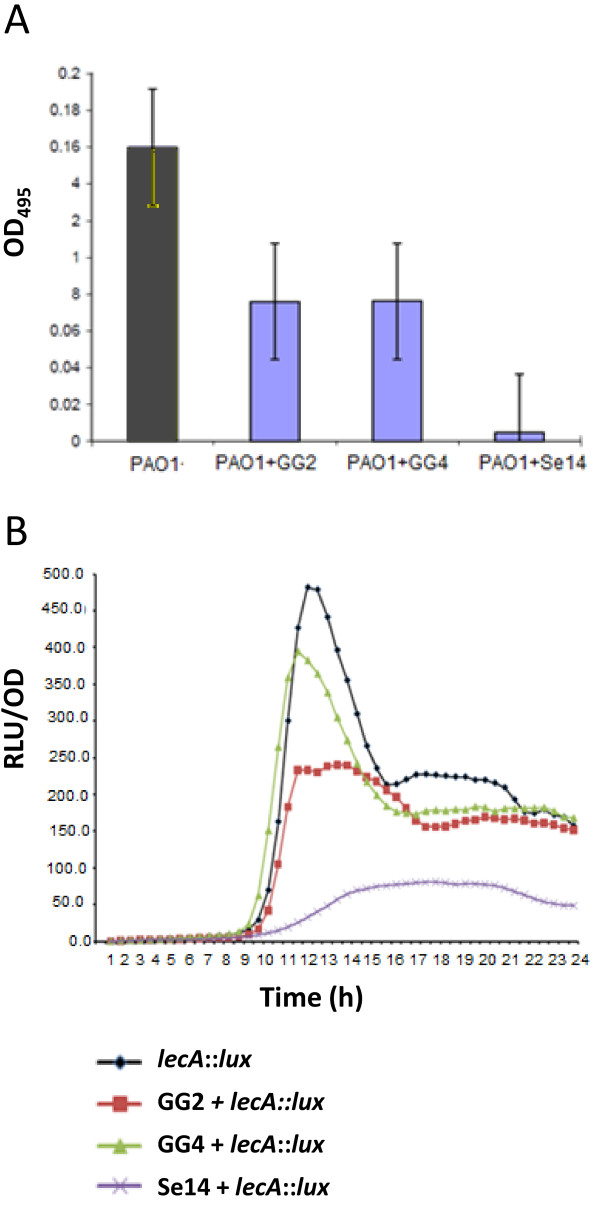
**Quenching of elastase production and *lecA *expression in *P. aeruginosa *by ginger rhizosphere strains**. (A) Elastase production by *P. aeruginosa *following monoculture (PAO1) or in co-culture with GG2 (PAO1+GG2), GG4 (PAO1+GG4) or Se14 (PAO1+Se14) at a starting inoculum ratio of 1:1 for 24 h. (B) Expression of a *lecA::lux *fusion following monoculture or co-culture of *P. aeruginosa *PAO1 with GG2, GG4 or Se14 at a starting inoculum ratio of 1:1 for 24 h. The data are presented as RLU/OD to account for any differences in growth.

To determine whether any of the three ginger rhizosphere bacterial isolates were capable of quenching virulence factor production in human (*P. aeruginosa*) and plant (*Er. carotovora*) pathogens which utilize different AHLs, we undertook co-culture experiments. Figure 4A shows that *Acinetobacter *GG2 and *Burkholderia *GG4 both reduced elastase production approximately two-fold when compared to the *P. aeruginosa *PAO1 control whereas the *Klebsiella *strain Se14 was the most effective, reducing elastase levels about 16-fold. None of the QQ bacteria inhibited the growth of *P. aeruginosa *which reached a similar viable count in co-culture as was attained in monoculture (data not shown). GG2 and Se14 both effectively reduced the expression of *lecA *in *P. aeruginosa *although GG4 had comparatively little effect (Figure 4B).

**Figure 5:**
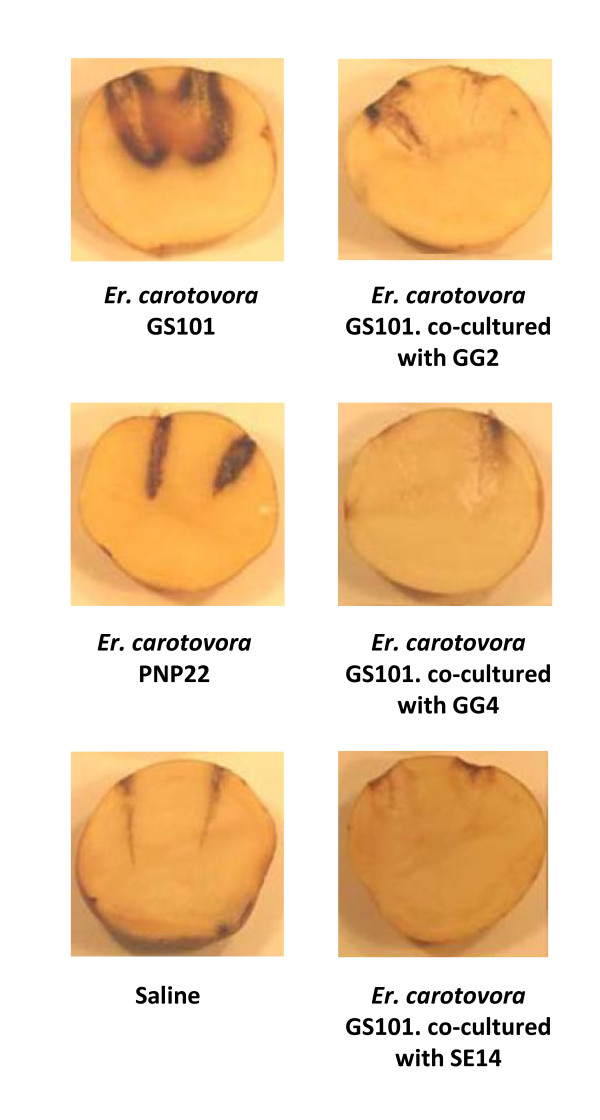
**Quenching of the pectinolytic activity of *Er. carotovora *by ginger rhizosphere strains**. The ability of GG2, GG4 and Se14 to reduce *Er. carotovora*-mediated soft rot in potato tuber tissues in co-culture was compared with the parent *Erwinia *strain in monoculture and, as negative controls, either the AHL-negative *Er. carotovora *mutant PNP22 or saline.

The QQ potential of GG2, GG4 and Se14 for attenuating the 3-oxo-C6-HSL-dependent pectinolytic activity of *Er*. *carotovora *was assessed *in planta *using a potato tuber infection model after viable count experiments confirmed that none of the strains affected the growth of *Er. carotovora *in co-culture experiments (data not shown). *Er*. *carotovora *causes substantial tissue necrosis when injected into the potato tuber but when co-cultured with any of the three ginger rhizosphere isolates, maceration of the potato tissue by the phytopathogen was greatly reduced (Figure 5).

## Discussion

In the present work, four bacterial morphotypes from the same ginger rhizosphere bacterial community were isolated and identified as a consequence of their ability to grow on an enrichment medium [14] containing 3-oxo-C6-HSL as the sole carbon and nitrogen source. BLAST search analyses of the 16S rDNA sequences identified the strains as belonging to the genera *Acinetobacter, Burkholderia, Klebsiella *and *Microbacterium*. In semi-quantitative whole cell assays, we evaluated the AHL-inactivating spectrum of the three Gram-negative isolates. The broadest range of activity was noted for *Klebsiella *strain Se14 which inactivated each of the 24 structurally diverse AHLs evaluated including the D-isomer of 3-oxo-C6-HSL. Similarly *Acinetobacter *strain GG2 exhibited a broad spectrum of activity but was less effective against short chain AHLs. In contrast, *Burkholderia *GG4 was inactive against the unsubstituted AHLs but was active against the 3-oxo-AHLs. Although AHL-degrading activity has not previously been characterized in the genus *Burkholderia*, a soil isolate from this genus capable of growing on AHLs as the sole nitrogen but not carbon source was reported by Yang *et al *[19]. This differs from *Burkholderia *strain GG4 which did not grow on 3-oxo-C6-HSL as a source of both carbon and nitrogen and probably came through the enrichment process as a consequence of AHL turnover by the other bacteria in the ginger rhizosphere community. Nevertheless, when GG4 was incubated with 3-oxo-C6-HSL in PBS buffer, GG4 reduced this AHL to the corresponding 3-hydroxy compound. Similar results were obtained for 3-oxo-C4-HSL and 3-oxo-C8-HSL as well as the D-isomer of 3-oxo-C6-HSL indicating that the activity was not AHL chain length dependent or stereospecific. This simple reduction of a 3-oxo-AHL to the corresponding 3-hydroxy compound is likely to impact on QQ. For example, in *Er. carotovora *where carbapenem antibiotic biosynthesis and exoenzyme production are regulated by 3-oxo-C6-HSL, the corresponding 3-hydroxy compound has only 1% of the activity of the 3-oxo-AHL [20]. For *P. aeruginosa*, the 3-hydroxy-C12-HSL was approximately 8-fold less active than the 3-oxo compound [21]. These data suggest that simple modification of the 3-oxo moiety is likely to substantially reduce the activity of 3-oxo-AHLs and to contribute to the QQ activity within a bacterial community. A similar oxido-reductase activity has been observed for a strain of *Rhodococcus erythropolis *isolated from the tobacco rhizosphere [22]. In contrast to *Burkholderia *strain GG4, this Gram positive bacterium (*R*. *erythropolis*) was unable to reduce 3-oxo-C6-HSL and required an AHL acyl chain of at least eight carbons [22]. However in common with GG4, the activity was only observed on incubation of 3-oxo-AHLs with whole, live bacterial cells as cell lysates were inactive [22].

For *Klebsiella *and *Acinetobacter*, AHL-inactivating activity has previously been noted by Park *et al *[11] and Kang *et al *[23], respectively. For the former, an AHL-degrading enzyme (AhlK) related to AhlD from *Arthrobacter *has been cloned and sequenced and by homology suggested to be a lactonase [11]. Here we have shown that the same gene is conserved in the *Klebsiella *ginger rhizosphere isolate Se14 and have demonstrated that the recombinant enzyme expressed in *E. coli *is indeed a lactonase with very broad AHL-inactivating activity including both short and long chain AHLs (with saturated or unsaturated acyl side chains of 4 to 14 carbons). These include *N*-(3-hydroxy-7-cis-tetradecanoyl)homoserine lactone (3-hydroxy-C_14:1_-HSL), an AHL which was originally termed the *Rhizobium *small bacteriocin [24] because it inhibits the growth of *Rhizobium leguminosarum *strains which carry a 'sensitivity locus' on Sym plasmids such as pRLJ1 [24]. 3-hydroxy-C_14:1_-HSL is also produced by soil bacteria such as *Pseudomonas fluorescens *[17].

*Acinetobacter *GG2 also degraded a wide range of short and long chain AHLs via a lactonase activity although we were unable to identify the gene involved. Although the mechanism of AHL degradation has not previously been determined in this genus, an *Acinetobacter *strain isolated from cucumber rhizosphere has been reported to degrade both C6-HSL and *N*-octadecanoyl homoserine lactone (C18-HSL) as well as the AHLs produced by a biocontrol strain of *Pseudomonas chlororaphis *and a phytopathogenic strain of *Burkholderia glumae *[23]. Interestingly, *Acinetobacter *GG2 not only degrades AHLs but also produces AHLs which we identified as 3-hydroxy-C12-HSL (major) and C12-HSL (minor). Previously Niu *et al *[25] showed that the human nosocomial pathogen, *Acinetobacter baumannii*, produces 3-hydroxy-C12-HSL and C12-HSL via the LuxI synthase, AbaI, the expression of which is AHL dependent. In *A. baumannii*, AHL-dependent QS appears to contribute to biofilm development since *abaI *mutants were less biofilm proficient than the parent strain [25].

*Acinetobacter *strains isolated from contact lenses have been reported not to produce any AHLs [26] whereas a study of 43 *Acinetobacter *strains isolated from both hospital patients and the environment and assayed using AHL biosensors showed that most of the strains examined produced AHLs although these were not chemically characterized [27]. Furthermore a comparative genome analysis of three different *Acinetobacter *strains from three different environments revealed the presence of a *luxIR*-type locus in a multidrug resistant clinical *A. baumannii *isolate which was disrupted by an insertion element in a sensitive strain isolated from human body lice but completely absent from a soil isolate [28].

In *Acinetobacter *GG2, 3-hydroxy-C12-HSL accumulated in the growth medium reaching a maximal level after 12 h before rapidly being degraded. This indicates GG2 tightly controls its own AHL production and turnover and suggests that sustained expression (or repression) of the QS target genes is not required in stationary phase. The coupling of AHL synthesis and degradation in the same bacterium has previously been noted for *Agrobacterium tumefaciens *which produces and degrades 3-oxo-C8-HSL during early stationary phase via a lactonase encoded by *attM *which is activated by starvation signals and the stress alarmone (p)ppGpp [29,30]. Similarly, a marine *Shewanella *strain which produces AHLs in late exponential phase degraded its long chain AHLs in stationary phase via both lactonase and acylase/amidase activities [31]. In polymicrobial biofilms, this *Shewanella *isolate interfered with AHL production in other bacteria and as a consequence, their ability to enhance the settlement of algal zoospores was compromised [31]. Here, we also found that the ginger rhizosphere *Burkholderia *isolate GG4 is not only capable of interfering with QS by reducing 3-oxo-AHLs to the corresponding 3-hydroxy compounds but also produces AHLs including 3-oxo-C6-HSL, C9-HSL and 3-hydroxy-C8-HSL. While most *Burkholderia *strains synthesize C6-HSL and C8-HSL [32,33], 3-hydroxy-C8-HSL production has only been confirmed in the pathogen, *Burkholderia mallei *[32] and tentatively identified in the environmental non-pathogenic *Burkholderia xenovorans *[33]. In *B. mallei*, C8-HSL and 3-hydroxy-C8-HSL are produced by two different AHL synthases (BmaI1 and BmaI3) [32]. In *Burkholderia *GG4, it remains to be established whether 3-hydroxy-C8-HSL is produced directly via a LuxI-type synthase or is a consequence of the reduction of 3-oxo-C8-HSL.

Bacteria such as GG2, GG4 and Se14 which produce and/or modify/degrade QS signals are likely to have a major impact on the properties of polymicrobial bacterial communities. Here we have shown that the ginger rhizosphere isolates were each capable of reducing virulence factor production in both *P. aeruginosa *and *Er. carotovora*. However, GG4 was unable to down-regulate *lecA *(which codes for the cytotoxic galactophilic lectin A [34]) expression probably as a consequence of its inability to reduce C4-HSL [35] in contrast to elastase which is predominantly LasR/3-oxo-C12-HSL dependent [36].

## Conclusions

Three different QQ bacteria associated with the ginger rhizosphere have been isolated and characterized as belonging to the genera *Acinetobacter *(GG2), *Burkholderia *(GG4) and *Klebsiella *(Se14). GG2 and Se14 exhibited the broadest spectrum of AHL degrading activity via lactonolysis while GG4 reduced 3-oxo-AHLs to the corresponding 3-hydroxy compounds. In GG2 and GG4, AHL-dependent QQ co-exists with AHL-dependent QS suggesting that these bacteria are likely to play a major role in determining the QS-dependent phenotype of the polymicrobial community from which they were isolated. This was confirmed by co-culture experiments in which all three rhizosphere bacteria attenuated virulence factor production in both a human and a plant pathogen without inhibiting growth of either pathogen.

## Methods

### Bacterial strains, growth media and culture conditions

The bacterial strains used in this study are listed in Table 2. Bacteria were routinely grown in Luria Bertani (LB) medium buffered when required with 50 mM 3-[*N-*morpholino] propanesulfonic acid (MOPS) to pH 6.8 to prevent alkaline hydrolysis of AHLs [8]. For the enrichment of QQ bacteria from the ginger rhizosphere, KG medium supplemented with 3-oxo-C6-HSL (500 μg/ml) was used [14]. *C*. *violaceum *CV026, *Er. carotovora *strains and the rhizosphere isolates were grown at 28°C, *E*. *coli *and *P. aeruginosa *strains at 37°C. When required, the *E. coli *growth medium was supplemented with ampicillin (100 μg/ml) and tetracycline (5 μg/ml). *C. violaceum *CV026 required kanamycin (30 μg/ml) and chloramphenicol (30 μg/ml).

**Table 2:** Strains used in the study

Strain	Description	Source/reference
***E. coli***		
DH5α	*recA endA1 hsdR17 supE4 gyrA96 relA1 ****Δ***(*lacZYA-argF*)U169 (*Φ80dlacZ****Δ****M15*)	[37]
pSB1075	*lasRlasl*' (*P*. *aeruginosa *PAO1)::*luxCDABE *(*Photorhabdus luminescens *[ATCC 29999]) fusion in pUC18 Amp^R^, AHL biosensor producing bioluminescence	[40]
pSB401	*luxRluxl*' (*Photobacterium fischeri *[ATCC 7744])::*luxCDABE *(*Photorhabdus luminescens *[ATCC 29999]) fusion; pACYC184-derived, Tet^R^, AHL biosensor producing bioluminescence	[40]
***C*. *violaceum***		
CV026	Double mini-Tn*5 *mutant derived from ATCC 31532, Kan^R^, Hg^R^, *cviI*::Tn*5 xylE*, plus spontaneous Str^R ^AHL biosensor, produces violacein pigment only in the presence of exogenous AHL	[15]
***Er. carotovora***		
		
GS101	AHL producing *Erwinia *strain, pectinolytic positive	[44]
PNP22	AHL-synthase mutant	[44]
***P. aeruginosa***		
PAO1	Prototroph	Lab collection
*lecA*::*lux*	*lecA*::*luxCDABE *genomic reporter fusion in PAO1	[35]
**Ginger rhizosphere-associated bacteria**		
*Acinetobacter *GG2	Ginger rhizosphere-associated bacterium	This study
*Burkholderia *GG4	Ginger rhizosphere-associated bacterium	This study
*Klebsiella *Se14	Ginger rhizosphere-associated bacterium	This study

### Enrichment procedures for bacteria degrading AHL from ginger rhizosphere

Ginger roots were collected at the Rimba Ilmu, University of Malaya (Malaysia). Soil was removed by rinsing thoroughly with sterile distilled water after which 1 g of rhizosphere tissue was added to 10 ml of KG medium, vortexed vigorously for 10 min, sonicated for 1 min and finally vortexed for 1 min. After which, 2 ml of this suspension was briefly centrifuged to remove root debris, re-centrifuged at 13,000 × *g *(5 min) after which the pellet was washed and resuspended in 2 ml of KG medium to give the final rhizosphere suspension. Then, 100 μl of this suspension was inoculated into 3 ml of KG medium containing 3-oxo-C6-HSL (500 μg/ml) and the cells were grown at 28°C with shaking at 220 rpm. After 48 h, a 5% (v/v) transfer was made to fresh, sterile KG medium and subsequent transfers made at 48 h intervals. After six enrichments the appropriately diluted cell cultures were plated onto LB agar and KG medium supplemented with 3-oxo-C6-HSL (50 μM) solidified with 1.5% (w/v) Bacto-Agar to isolate individual colonies.

### DNA manipulation

Genomic DNA and plasmid extraction, manipulation and competent cells were prepared using standard methods [37]. Treatment of PCR mixtures without DNA template was performed as previously described [38]. PCR mix (Promega, UK) was used to amplify 16S rDNA with the universal primers 27F and 1525R (Table 3). PCR conditions, cloning and sequencing of the PCR products were carried out as previously described [14]. DNA sequences were analysed with the Lasergene computer package (DNAstar) in combination with the BLAST programs available from NCBI http://www.ncbi.nlm.nih.gov/ while phylogenetic analyses were performed as previously described [14]. The *ahlK *gene was amplified from *Klebsiella *Se14 by PCR using the primers KF and KR (Table 3). A single band of 0.85 kb was amplified and ligated to pGEM-T Easy and introduced into *E. coli *DH5α. A positive clone exhibiting QQ activity was sequenced.

**Table 3:** Oligonucleotide Primers

Name	Sequence	Reference
16S rDNA forward primer 27F	5'-AGAGTTTGATCMTGGCTCAG-3'	[14]
16S rDNA reverse primer 1525R	5'-AAGGAGGTGWTCCARCC-3'	[14]
KF forward primer	5'-CTGAATTCCTGAGTCAGGCTA-3'	[11]
KR reverse primer	5'-TTGAATTCTCAGCGAGGAATGAT-3'	[11]

### Synthesis of AHLs and related compounds

AHLs including the D-isomer of 3-oxo-C6-HSL were synthesized, purified and characterized as previously described [20,39].

### AHL-inactivation assays

GG2, GG4 and Se14 were grown overnight at 28°C with shaking (220 rpm) in LB medium to approximately 10^9 ^cfu/ml, cells (100 ml) were collected by centrifugation, washed and resuspended in 100 ml of PBS (100 mM, pH 6.5). AHLs were evaporated to dryness in a suitable tube and rehydrated with cell suspension providing a final AHL concentration of 1 μM (for biosensor activation assays) or 50 μM (for HPLC analysis). The reaction mixture was incubated for up to 24 h at 28°C with gentle shaking. To stop the reaction, an equal volume of ethyl acetate was added, after which the AHLs were extracted with ethyl acetate. Any residual AHLs were detected using the *lux*-based biosensors *E*. *coli *[pSB401] or *E*. *coli *[pSB1075] for long and short chain AHLs, respectively, [40] using a microtitre plate bioassay based on that described by Reimmann *et al *[41]. Bioluminescence in the microtitre plate wells was visualized using Luminograph LB980 photon video camera (Berthold).

To determine whether AHLs were being inactivated by lactonolysis, i.e. by the formation of the corresponding *N*-acylhomoserine compound, the method described by Yates *et al *[8] was used. This is based on acidification of the reaction mixture to pH 2 with HCl (10 mM) to promote recyclization of the homoserine lactone ring.

### HPLC analysis of AHLs and AHL-degradation products

HPLC analysis of AHLs and their degradation products was performed as described before [17,20] on an analytical C_8 _reverse-phase preparative HPLC column (Kromasil C8; 250 × 4.6 mm) attached to a photodiode array (PDA) system (Waters 996 PDA system operating with a Millennium 2010 Chromatography Manager, Waters, England) and eluted with acetonitrile/water isocratic or gradient combinations as described before [17].

### Identification of AHLs

AHLs were unequivocally identified by LC-MS/MS as described before [17,42] using enhanced product trap experiments (EPI) triggered by precursor ion scanning between the *m/z *range 150-500 and in particular for the fragment ion *m/z *102 which is characteristic for the homoserine lactone ring moiety. The EPI spectra (*m/z *range 80-400) containing a fragment ion at *m/z *102 were compared for the retention time and spectral properties to a series of corresponding synthetic AHL standards. The 3-hydroxy-AHLs were identified by comparison with a synthetic standard based on the LC retention times, the MS-MS fragmentation product ions ([M+H-H_2_O] and *m/z *102). 3-hydroxy-AHLs characteristically lose a water molecule during MS fragmentation generating a characteristic ion of [M-18] [17,43].

### *P. aeruginosa *QQ co-culture assays

The ability of ginger rhizosphere isolates to attenuate *P*. *aeruginosa *QS-regulated virulence determinants (elastase and lectin A) were determined by growing cultures of *P*. *aeruginosa *PAO1, GG2, GG4 and Se14 separately at 28°C for 24 h with shaking (220 rpm), normalizing at an OD_600 _of 1.0 followed by co-culturing at a 1:1 ratio. Total viable cell counts were carried out to ensure that neither organism significantly reduced the growth of the other.

The elastolytic activity of *P*. *aeruginosa *was determined as described before using elastin-Congo red (ECR) as substrate. Briefly, 100 μl of cell free bacterial spent culture supernatants from both mono-culture and co-culture experiments were added separately to 900 μl ECR buffer (100 mM Tris [pH 7.5], 1 mM CaCl_2_) containing 20 mg of ECR and incubated with shaking at 37°C for 3 h. Insoluble ECR was removed by centrifugation at 7,000 × *g *for 5 min. The absorbance of the supernatant was determined at OD_495_. The expression of *lecA *was determined using a *P*. *aeruginosa lecA*::*lux *reporter strain [35] in a 96-well microtitre plate using an automated combined luminometer/spectrometer (Anthos Labtech LUCYI). Briefly, 200 μl of a 1:500 dilution of an overnight culture of the *P*. *aeruginosa lecA*::*lux *reporter and either GG2, GG4 or Se14 (100 μl from each culture in co-culture experiments, or 200 μl in monoculture experiments) were loaded into the plate wells and luminescence and OD_495 _were determined every 30 min for 24 h. Each co-culture experiment was carried out in triplicate and repeated at least twice.

### Quenching of the pectinolytic activity of *Er. carotovora*

Inhibition of the pectinolytic activity of *Er. carotovora *was carried out with modification as described before [44,45] using potato tubers. Tubers were washed, sterilized with 70% v/v ethanol, then extensively rinsed with sterile water and finally dried under sterile conditions. Bacterial cells were grown overnight at 28°C in LB, washed, resuspended and diluted in sterile saline to OD_600 _of 1.0. Bacterial suspensions (*Er. carotovora *GS101 or the *Er. carotovora *AHL-synthase mutant PNP22 [44]) (negative controls), monocultures or co-cultured with GG2, GG4 or Se14 were introduced directly into the tubers using a 200-μl tip fitted on a micropipette. Tubers were incubated at 25°C, 90% humidity for 3 days. The results of the inoculation were assessed by visual inspection after slicing the tubers.

## Abbreviations

AHL: *N*-acylhomoserine lactones; 3-oxo-C6-HSL: *N*-(3-oxo-hexanoyl)homoserine lactone; C4-HSL: *N*-butanoylhomoserine lactone; C5-HSL: *N*-pentanoylhomoserine lactone; C6-HSL: *N*-hexanoyl homoserine lactone; C7-HSL: *N*-heptanoylhomoserine lactone; C8-HSL: *N*-octanoylhomoserine lactone; C9-HSL: *N*-nonanoylhomoserine lactone; C10-HSL: *N*-decanoylhomoserine lactone; C11-HSL: *N*-undecanoylhomoserine lactone; C12-HSL: *N*-dodecanoylhomoserine lactone; C14-HSL: *N*-tetradecanoylhomoserine lactone; 3-hydroxy-C4-HSL: *N*-(3-hydroxybutanoyl)homoserine lactone; 3-hydroxy-C6-HSL: *N*-(3-hydroxyhexanoyl)homoserine lactone; 3-hydroxy-C8-HSL: *N*-(3-hydroxyoctanoyl)homoserine lactone; 3-hydroxy-C10-HSL: *N*-(3-hydroxydecanoyl)homoserine lactone; 3-hydroxy-C12-HSL: *N*-(3-hydroxydodecanoyl)homoserine lactone; 3-hydroxy-C14-HSL: *N*-(3-hydroxytetradecanoyl)homoserine lactone; Δ^9^-3-hydroxy-C14-HSL: *N*-(3-hydroxy-9-*cis*- tetradecanoyl)homoserine lactone; Δ^10^-3-hydroxy-C14-HSL: *N*-(3-hydroxy-10-*cis*-tetradecanoyl)homoserine lactone; Δ^11^-3-hydroxy-C14-HSL: *N*-(3-hydroxy-11-*cis*-tetradecanoyl)homoserine lactone; Δ^13^-3-hydroxy-C14-HSL: *N*-(3-hydroxy-13-*cis*-tetradecanoyl)homoserine lactone; CFU: colony forming units, QS: quorum sensing, QQ: quorum quenching;

## Authors' contributions

KGC carried out the experiments other than LC-MS/MS. SA, KM helped draft the manuscript. SRC supervised the AHL syntheses and interpreted the MS spectra. MC established the HPLC method. CLK, CKS and PW conceived the study, helped in the biological interpretation, and drafted the manuscript. All authors read and approved the final manuscript.

## Supplementary Material

Additional file 1 **Mass spectra AHLs produced by GG4**. Extracts from spent culture supernatants of GG4 were analysed by mass spectrometry. The peak ion at *m/z *102 is characteristic of the homoserine lactone ring (A, B, E and F). By comparison with the corresponding synthetic standards (C, D, G and H) the precursor ion at *m/z *214.2 and fragment ion at *m/z *113.0 suggest the presence of 3-oxo-C6-HSL (A); the precursor ion at *m/z *228.2 and fragment ion *m/z *109.1 are indicative of C8-HSL (B); the precursor ion at *m/z *226.2 [M-H_2_O] and fragment ion *m/z *125.1 are indicative of 3-hydroxy-C8-HSL (E); the precursor ion at *m/z *242.2 and fragment ion of *m/z *142.2 are indicative of C9-HSL (F). AU: Absorbance unit. Click here for file
