# Supplementary figures and images for: Characterization of N-acylhomoserine lactone-degrading bacteria associated with the Zingiber officinale (ginger) rhizosphere: Co-existence of quorum quenching and quorum sensing in Acinetobacter and Burkholderia
Source: BMC Microbiol. 2011 Mar 8;11:51. doi: 10.1186/1471-2180-11-51 (PMC3062576; doi:10.1186/1471-2180-11-51)

## Slide 1
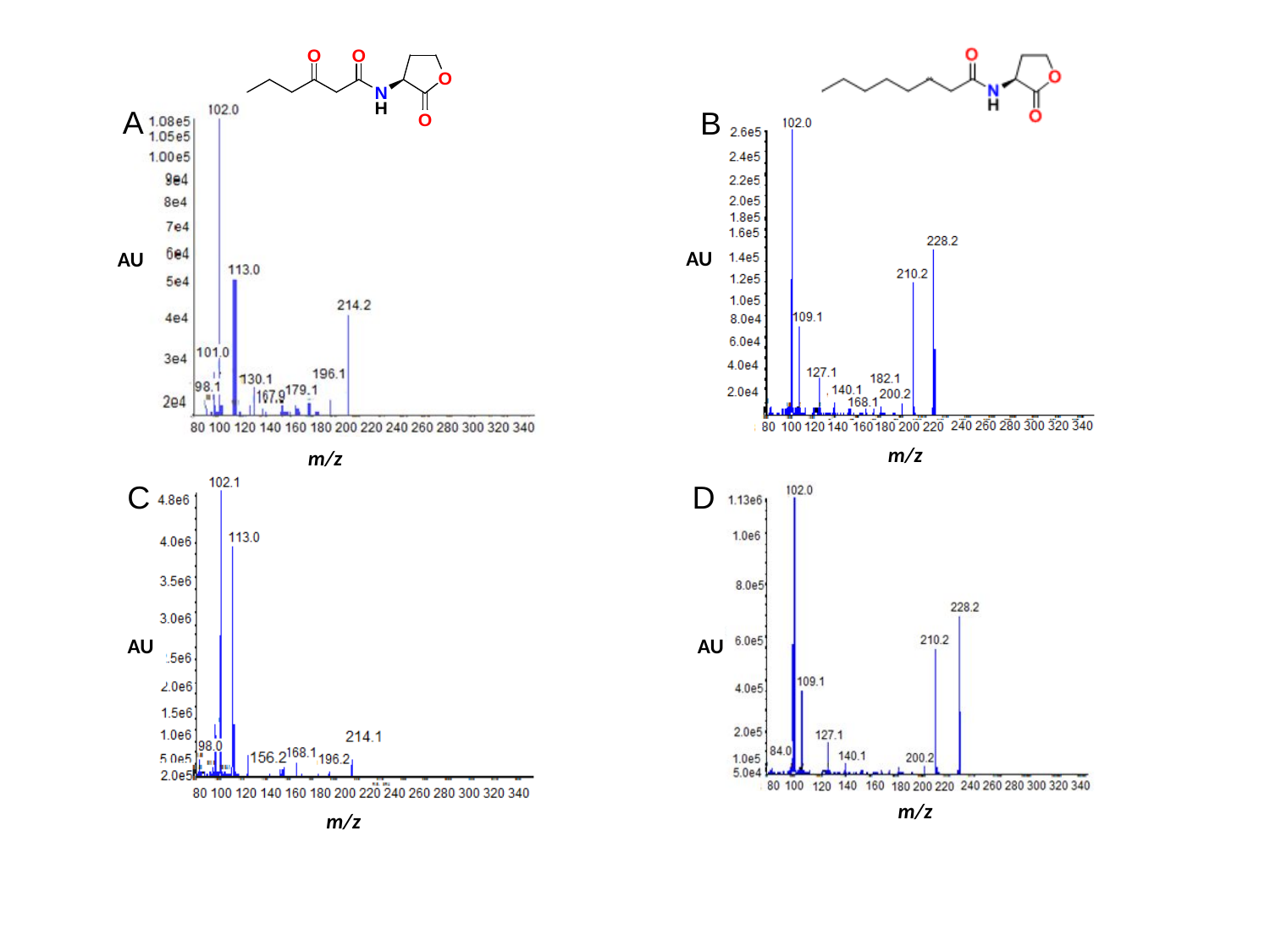

A
B
AU
AU
m/z
m/z
C
 D
AU
AU
m/z
m/z

## Slide 2
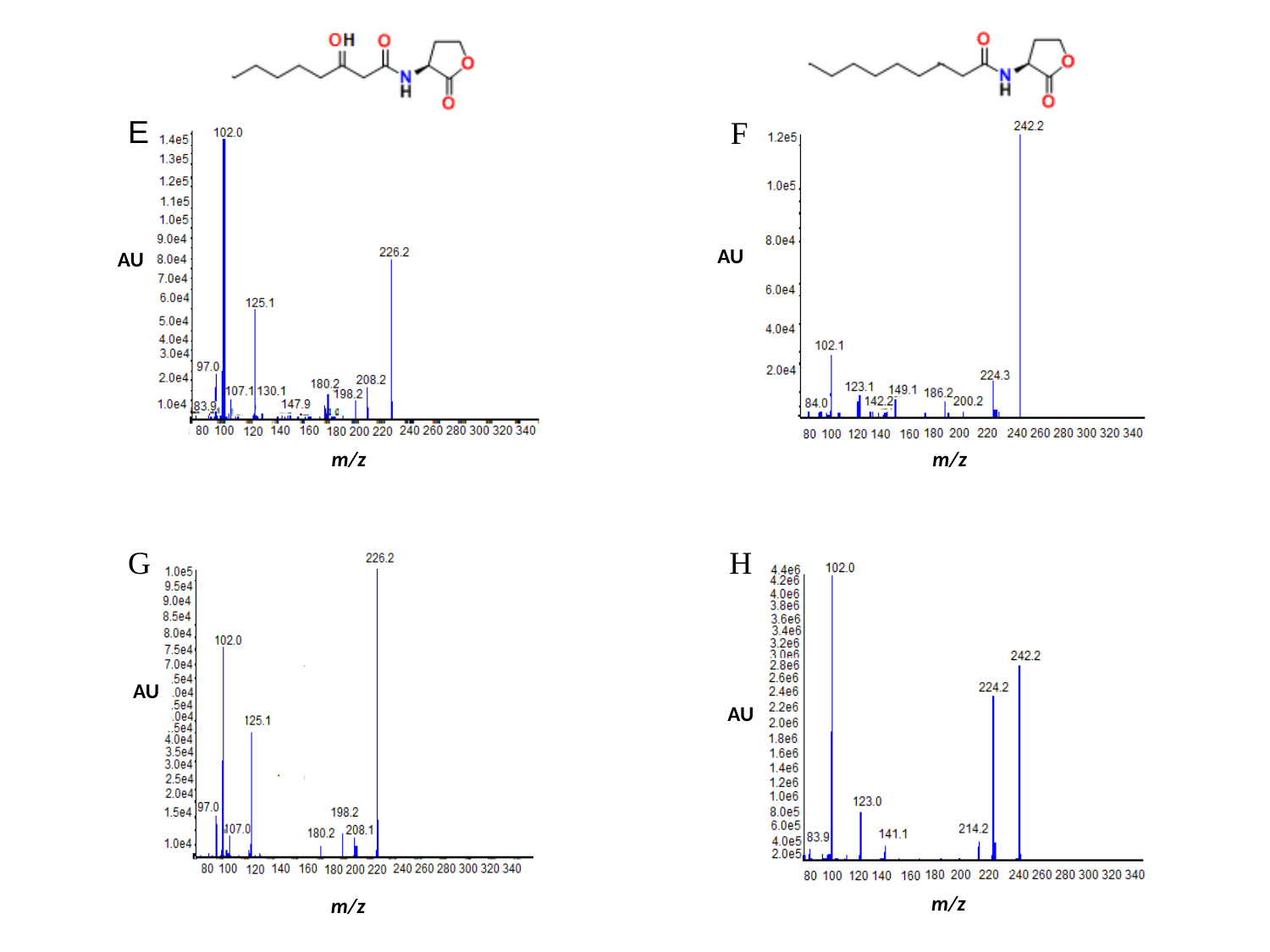

E
F
AU
AU
m/z
m/z
G
H
AU
AU
m/z
m/z

Supplement: Additional file 1 — Mass spectra AHLs produced by GG4. Extracts from spent culture supernatants of GG4 were analysed by mass spectrometry. The peak ion at m/z 102 is characteristic of the homoserine lactone ring (A, B, E and F). By comparison with the corresponding synthetic standards (C, D, G and H) the precursor ion at m/z 214.2 and fragment ion at m/z 113.0 suggest the presence of 3-oxo-C6-HSL (A); the precursor ion at m/z 228.2 and fragment ion m/z 109.1 are indicative of C8-HSL (B); the precursor ion at m/z 226.2 [M-H2O] and fragment ion m/z 125.1 are indicative of 3-hydroxy-C8-HSL (E); the precursor ion at m/z 242.2 and fragment ion of m/z 142.2 are indicative of C9-HSL (F). AU: Absorbance unit. [file 1471-2180-11-51-S1.PPT]
